# A Case of Almost Total Destruction of the Iris; Recovery with Perfect Vision

**Published:** 1890-04

**Authors:** R. O. Cotter

**Affiliations:** Macon, Georgia


### A CASE OF ALMOST TOTAL DESTRUCTION OF THE IRIS—RECOVERY WITH PERFECT VISION.

BY R. O. COTTER, M. D., MACON, GEORGIA.

The following case is by far the most remarkable one that I have ever seen, and with the literature at my command, I can find no record of any similar case. It shows quite conclusively that it is indeed true that, even with all, or nearly all, of the iris removed there can be good accommodation left. It also will disprove the truth of the assertion which the ardent advocates of the new ( ? ) operation for extracting of cataract without iridectomy make in defense of the new fad in the operation, which is really not a new operation, but is simply a revival of the old simple extraction of thirty or thirty-five years ago.

They claim that an ordinary iridectomy cuts off considerable of the patient's acuity of vision. The case I report will show what vision remained with three-fourths or more of the iris gone. By way of digression I will say, I have a report of seven cataract extractions made without iridectomy within the past two months. I shall publish this soon, and in spite of the fact that only one case was a failure, I will present what I consider as very strong objections to the operation without iridectomy.

B. C., age 36, farmer, was struck late last June in the right eye by a sharp, flat piece of flint rock. He suffered intensely, and on July 1st he came to me for treatment. I found that the piece of rock had penetrated the cornea close to the iris, at the temporal side. It was so large as to almost completely fill up the upper half of the anterior chamber, and was firmly imbedded and adherent to the iris. Although I freely used a 10 per cent. solution of cocaine, it made hardly any impression upon the pain of the operation.

The corneo-sclerotic junction was incised with a cataract knife, just as if for a cataract extraction, and the iris was found to be so bruised, lacerated and involved in the wound from the foreign body that I had to remove fully three-fourths of the iris in order to extract the piece of rock. There seemed hardly any prospect of vision to be secured and my idea was to give him relief from the pain and prevent, if possible, total destruction of the ball. The ragged edges of the wounded iris were carefully trimmed, a 2 grain solution of atropia was directed to be used in the eye for a few days, warm water applications to the eye advised and the patient sent home.

To day (Feb'y 13th) he was in town and called on me. I was greatly surprised at his assertion that he could see with the eye as good as he ever could. The eye had healed entirely. He has only a narrow strip of iris in the lower segment. There is hardly three-fourths of the iris left. I tested his vision and accommodation carefully. His vision is perfectly normal, being 20-20. He reads Jaeger No. 1 type (the very smallest) easily at the usual reading distance, with ease.

